# Peroxisome dynamics and inter-organelle interactions in neuronal health and disease

**DOI:** 10.3389/fnmol.2025.1603632

**Published:** 2025-06-20

**Authors:** Ruth E. Carmichael

**Affiliations:** Department of Biosciences, Faculty of Health and Life Sciences, University of Exeter, Exeter, United Kingdom

**Keywords:** peroxisome, neuron, membrane dynamics, membrane contact sites, PEX11β, MFF, neurodegeneration

## Abstract

Peroxisomes are essential organelles, present in all nucleated cells, with key roles in lipid and redox homeostasis. They are important for maintaining healthy cell function, with defects in peroxisome biogenesis and/or metabolism leading to disease. Notably, patients with peroxisomal diseases exhibit predominantly neurological phenotypes, and peroxisomes are observed to be altered in a range of neurodegenerative conditions, highlighting the crucial roles they play in the brain. While most studies so far have focused on the contribution of peroxisomal metabolism, it is becoming apparent that many different aspects of peroxisome biology are necessary for healthy neural function. Peroxisomes are highly dynamic, responding to cellular needs with changes in number, shape and distribution. Furthermore, they do not act in isolation but instead interact and cooperate with a range of organelles to carry out their roles. This review summarizes our current knowledge on the importance of peroxisome dynamics and inter-organelle interactions in neuronal function and dysfunction. It considers their impact on neuronal physiology, and discusses the evidence that defects in these processes are associated with neurological pathophysiology and may thus represent a novel therapeutic target for treating diseases affecting the nervous system. Finally, the review outlines the current knowledge gaps relating to the mechanisms by which peroxisome dynamics and inter-organelle interactions influence neuronal (dys)function, proposing potential new research directions to address these and further our understanding of the multi-faceted roles peroxisomes play in brain health and disease.

## 1 Introduction

Peroxisomes are ubiquitous oxidative organelles that are essential for healthy cellular function, performing crucial roles in diverse metabolic and signaling processes including α- and β-oxidation of complex fatty acids [e.g., very long-chain fatty acids (VLCFAs)], synthesis of ether-phospholipids, maintenance of cellular redox balance and pathogen defense (Kumar et al., 2024). One key feature of peroxisomes is their remarkable plasticity in response to stimuli, being able to dynamically and rapidly alter their number, morphology, function and distribution according to cellular demands (Carmichael and Schrader, 2022). They are also very “social” organelles, cooperating extensively with other intracellular compartments to fulfill their functions, including in lipid metabolism and reactive oxygen species (ROS) homeostasis (Sargsyan and Thoms, 2019; Silva et al., 2020). Neurons are highly excitable post-mitotic cells with large surface area to volume ratios and intricate polarized/compartmentalized morphologies, needing constant maintenance, protection and energy generation to support action-potential conductance and synaptic transmission (Steward, 2000). The high demands imposed by these metabolically intensive processes increases the need for organelles, including peroxisomes, to act flexibly and efficiently, both individually and as part of a wider inter-organelle network.

Peroxisomes are undoubtedly of crucial importance in the nervous system, as evidenced by the severe neurological alterations and phenotypes of patients with peroxisomal disorders (Wanders et al., 1988). Peroxisomes contribute to key metabolic processes that impact on brain function, for example, the synthesis of ether-phospholipids/plasmalogens, which are a major component of myelin sheath lipids, as well as the key brain lipid docosahexaenoic acid (DHA), which regulates membrane properties and synapse number/function (Ferdinandusse et al., 2001; Berger et al., 2016). While this review will focus exclusively on the role of peroxisomes in neurons, it is important to note that peroxisomes are also abundant and crucial in all types of glial cells, including astrocytes, microglia and oligodendrocytes (Deb et al., 2021). Mouse models lacking functional peroxisomes in all central nervous system (CNS) cells display neurological phenotypes including demyelination and axonal damage that are not so clearly observed in neuron-specific knock-out models (Hulshagen et al., 2008; Bottelbergs et al., 2010). Furthermore, CNS-wide, but not neuron-specific, loss of peroxisomal function results in metabolic alterations in brain tissue (Bottelbergs et al., 2010), suggesting that lipid metabolism is likely to be a major function of peroxisomes in glia [e.g., to support CNS energy homeostasis (Asadollahi et al., 2024)], with neuronal peroxisomes possibly performing more specialized roles *in-vivo*. This highlights the challenges of unraveling the relative contribution of peroxisomes in these different cell types in CNS pathologies, which is important to understand the complexity of neurological/neurodegenerative disorders. To dissect the functions of peroxisomes in neurons specifically, neuronal cultures have proved an important *in-vitro* tool, but likely display more striking effects without the compensation/support from glial peroxisomes that would be seen in the *in-vivo* context.

Research so far has mostly concentrated on the role of intraperoxisomal metabolism in the brain, however other aspects of peroxisomal biology are emerging as key determinants of neurological function. This review will provide an overview of our current knowledge on the role of peroxisome dynamics, and their interplay with other organelles, in neuronal cells. It will address how these processes maintain healthy neuronal function, as well as how they are dysregulated in disease states, and how this may be contributing to pathology. Finally, the review will outline the outstanding questions in the field, setting the scene for future research avenues concerning the multi-faceted roles of peroxisomes in neuronal biology.

## 2 Peroxisome dynamics in neuronal cells

**FIGURE 1:**
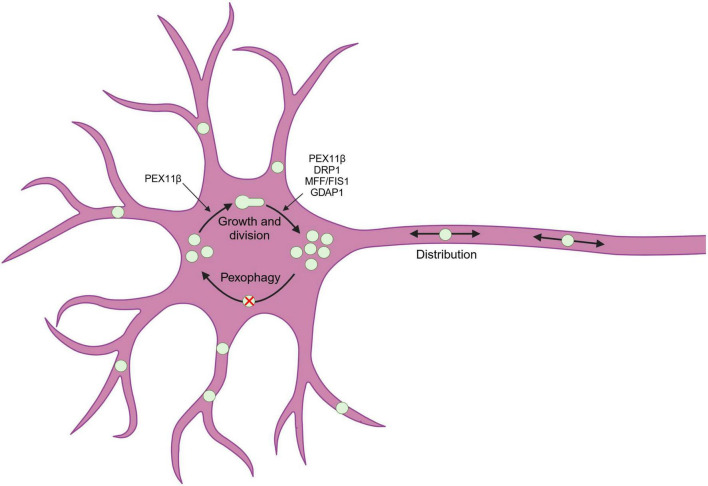
Peroxisome dynamics in neuronal cells. Peroxisomes (green circles) can dynamically change their number in response to intra- and extracellular signals. Peroxisomes can proliferate via growth and division, which starts with PEX11β-mediated deformation and elongation of the membrane, which then divides to yield multiple daughter peroxisomes in a process requiring the proteins DRP1, MFF/FIS1, PEX11β, and (in neurons) GDAP1. Peroxisomes are also present in the soma and neurites, and can be dynamically trafficked along neuronal processes. Created in BioRender (https://BioRender.com/e8hb9td).

Peroxisomes are highly dynamic across eukaryotic cells from yeast to man, rapidly adapting in response to stimuli. One key way that peroxisomes respond to their environment is through changes in their total volume within the cell, which will alter their overall metabolic capacity (Schrader et al., 2016). In mammalian cells, peroxisome proliferation is usually mediated via an increase in peroxisome number, which can occur through two possible mechanisms: *de novo*, where new peroxisomes are thought to form from endoplasmic reticulum (ER) and mitochondria-derived pre-peroxisomal vesicles (Hoepfner et al., 2005; Sugiura et al., 2017); and growth and division, where new peroxisomes arise from pre-existing ones (Delille et al., 2010; Smith and Aitchison, 2013). During the growth and division cycle (Figure 1), peroxisomes also undergo dynamic morphological changes, with the peroxisomal membrane deforming and elongating, before constricting and ultimately undergoing scission to yield multiple daughter peroxisomes (Carmichael and Schrader, 2022). The initial membrane remodeling is mediated by the peroxisomal membrane protein PEX11β, which contains an N-terminal amphipathic helix that can interact with and deform the outer leaflet (Opaliński et al., 2011; Yoshida et al., 2015). The constriction/division step requires MFF (mitochondrial fission factor) and/or FIS1 (mitochondrial fission 1), tail-anchored membrane proteins shared with mitochondria, that recruit the fission GTPase DRP1 (dynamin-related protein 1) to the peroxisomal membrane, where it oligomerizes around the organelle (Koch et al., 2005; Gandre-Babbe and van der Bliek, 2008; Itoyama et al., 2013). As well as membrane elongation, PEX11β also promotes fission by stimulating the GTPase activity of DRP1 which drives the conformation change necessary to constrict and ultimately divide the peroxisomal membrane (Williams et al., 2015). Furthermore, a neuron-enriched peroxisomal/mitochondrial tail-anchored membrane protein, GDAP1 (ganglioside-induced differentiation-associated protein 1), promotes peroxisomal fission in a DRP1- and MFF-dependent manner (Huber et al., 2013). To counteract proliferation and reduce peroxisome number when required, for example as a quality control mechanism when peroxisomes become damaged, peroxisomes can be selectively degraded by a process known as pexophagy, which requires the autophagic machinery (Figure 1; Eberhart and Kovacs, 2018).

**FIGURE 2:**
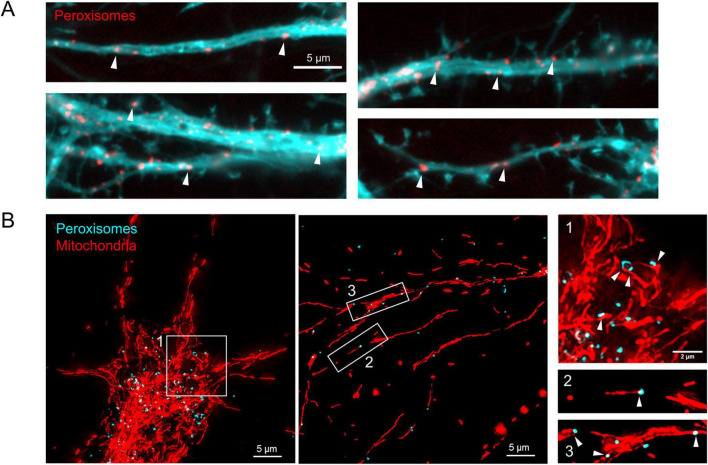
Peroxisome distribution and interactions with mitochondria in neurons. Primary hippocampal neuronal cultures prepared from E18 rats, transfected at DIV12 (12 *days in-vitro*) and imaged at DIV17. **(A)** Peroxisomes are observed in neurites (indicated by arrowheads) of cultured hippocampal neurons. Cells were co-transfected with cytosolic GFP (cyan) to fill an individual cell and its processes, and mScarlet-PMP (targeted to the peroxisomal membrane, red). **(B)** Peroxisomes and mitochondria are in close proximity (indicated by arrowheads) in both the soma (1) and neurites (2,3) of cultured hippocampal neurons. Peroxisomes were labeled by expressing GFP-PTS1 (targeted to the peroxisomal matrix, cyan), and mitochondria were labeled by expressing mito-dsRed (targeted to the mitochondrial outer membrane, red).

In addition to changes in number/volume and morphology, peroxisomes are also dynamic with respect to their distribution in the cell, which is particularly pertinent in neurons due to their polarized and compartmentalized nature (Figure 1). Under basal conditions, peroxisomes are mainly localized to the soma and proximal dendrites (Figure 2A; Wang et al., 2018), but their distribution depends on microtubule-dependent long-range movement (Schrader et al., 1996; Castro et al., 2018) as well as interactions with other organelles, e.g., tethering to the ER (see section 3.1) (Costello et al., 2017a; Covill-Cooke et al., 2021).

### 2.1 Peroxisome dynamics maintain neuronal physiology

The dynamic plasticity of peroxisomes is clearly crucial for healthy neuronal development and function, as evidenced by the neurological/neurodevelopmental phenotypes associated with diseases affecting proteins involved in peroxisome proliferation (see section 2.2.1). However, compared to mitochondria, we are only just starting to reveal the mechanisms by which peroxisome dynamics contribute to physiological neuronal processes, which are discussed below.

#### 2.1.1 Peroxisome dynamics during neuronal differentiation

Early electron microscopy studies demonstrated the presence of peroxisomes positive for the H_2_O_2_-decomposing matrix enzyme catalase in differentiating neurons throughout the cerebrum and cerebellum in rodents and humans. In both cases, neuronal peroxisome number increased during pre- and early post-natal development then decreased in mature neurons (Arnold and Holtzman, 1978; Houdou et al., 1991), reflected in a striking and progressive decrease in peroxisomal protein levels at 15 and 49 days after birth, compared to 2 days after birth, in all regions of mouse brain investigated (Ahlemeyer et al., 2007). An increase in peroxisome number was also observed during differentiation of the neuroblastoma cell line SH-SY5Y as well as human induced pluripotent stem cells (iPSCs) into neuronal cells *in-vitro* (Borisyuk et al., 2025). Altogether, this upregulation of peroxisome numbers during neurodevelopment specifically implies a temporary need for increased peroxisomal function during certain stages of differentiation, e.g., for lipid metabolism or ROS homeostasis, which is no longer so crucial once neurons reach maturity. However, how, why and when peroxisomes support neuronal differentiation has yet to be determined.

Furthermore, several studies have shown the “master regulator” of peroxisome proliferation, PEX11β, to be important in neuronal differentiation. Primary cortical cultures generated from homozygous *Pex11*β knock-out embryos, which can then develop *in-vitro*, displayed reduced protein levels of synaptophysin, a marker of synaptogenesis, after 7 days, as well as MAP2 (microtubule-associated protein 2), a marker of mature neuronal identity, compared to wild-type. Combined with reduced network formation/branching in these knock-out cultures, this indicates PEX11β is required for timely neuronal differentiation, as neuron development is delayed in its absence (Ahlemeyer et al., 2012). Similarly, inducible *Pex11*β knock-down suppressed differentiation of both mouse and human embryonic stem cells (ESCs) into neural progenitor cells and mature neuronal cells, as assessed by marker protein expression and reduced neurite outgrowth. In both cases, reduced expression of peroxisomal genes was also observed (Esmaeili et al., 2016, 2024). Crucially, pretreatment of mouse ESCs with the PPAR (peroxisome proliferator-activated receptor) γ agonist pioglitazone, which would be predicted to upregulate peroxisome numbers/protein expression, partially ameliorated the decreased differentiation seen upon *Pex11*β knock-down (Esmaeili et al., 2016). Together, this implicates PEX11β-dependent peroxisome proliferation as a driver of neuronal differentiation. It is notable that patients with a defect in PEX11β (see section 2.2.1) have not been reported to display prominent developmental brain phenotypes (Carmichael et al., 2022), which may represent species-specific differences, or compensation by the mother’s metabolism *in-utero*. The latter would therefore suggest that the reduced differentiation seen in PEX11β-deficient neuronal cultures is due to a lack of peroxisomal metabolites during crucial developmental stages.

#### 2.1.2 Peroxisomes dynamically alter to protect neurons under stress conditions

Peroxisomes are key organelles in regulating lipid and redox homeostasis, acting as essential adaptors to allow the cell to respond to metabolic and environmental stress (He et al., 2021). This protective role of peroxisomes under stress conditions could be one reason that peroxisome dynamics are so crucial in neurons—as post-mitotic cells, cell death has far more severe consequences when cells cannot be replaced by proliferation. Oxidative stress induces peroxisome multiplication in several post-mitotic cell types, including hair cells and ganglion neurons of the cochlea (Delmaghani et al., 2015). Forty eight hours after exposing mice to loud noise, which generates ROS in the cochlea, peroxisome numbers were increased in both inner and outer hair cells as well as in the dendrites of primary auditory neurons. Conversely, mice lacking the peroxisome-associated protein pejkavin displayed a decrease in peroxisome number in inner hair cells after sound exposure, resulting in hypervulnerability to noise-induced hearing loss and demonstrating the protective effect of peroxisome proliferation in maintaining ROS homeostasis (Delmaghani et al., 2015). A subsequent study showed that a rapid initial phase of pejkavin-dependent pexophagy in noise-exposed inner hair cells was necessary to “prime” the subsequent peroxisome proliferation, illustrating the importance of tight bidirectional control of peroxisome numbers during stress responses (Defourny et al., 2019).

Supporting the upregulation of peroxisome number as a protective mechanism against oxidative stress, peroxisome biogenesis is also stimulated in ischemic neurons. In brain tissue from a mouse model of ischemia, the total volume of immunolabelled peroxisomes was significantly increased in the peri-infarct area 24 hours after reperfusion, with similar results observed in cultured cortical neurons exposed to oxygen-glucose deprivation (OGD) (Young et al., 2015). The peroxisome proliferation in the peri-infarct region of ischemic brain was accompanied by increased catalase expression, while catalase inhibition increased cell death following OGD, indicating the increase in peroxisome number serves to bolster the cell’s antioxidative response to ischemia. Accordingly, upregulating peroxisome number via a PPARα agonist decreased, while downregulating peroxisome proliferation by DRP1 silencing increased, cell death induced by OGD in culture (Young et al., 2015).

#### 2.1.3 Lessons from mitochondrial dynamics in neurons

Much attention has been focused on the role of the shared mitochondrial/peroxisomal dynamics proteins DRP1, MFF and FIS1 in neuronal development and function, however the effects are usually attributed to their regulation of mitochondrial dynamics (Flippo and Strack, 2017; Seager et al., 2020; Baum and Gama, 2021), while the consequences for peroxisome dynamics are often overlooked. Nonetheless, these studies provide compelling evidence for the importance of regulated organelle dynamics in healthy neuronal function. For example, mitochondrial morphology is notably different in axons compared to dendrites (and even within axonal/dendritic compartments in a single cell), with axonal mitochondria being much smaller than those in dendrites, which are longer and more tubular (Popov et al., 2005; Faitg et al., 2021). The reduced size of mitochondria in axons in cortical pyramidal neurons depends on MFF-dependent fission and is thought to promote axon branching and neurotransmitter release by preventing excessive calcium buffering at presynapses (Lewis et al., 2018). Conversely, dendritic mitochondrial morphology may be more dependent on FIS1, which is more abundant on dendritic compared to axonal mitochondria. FIS1 knock-down counterintuitively reduced mitochondrial size while increasing motility, and led to increased dendrite branching but reduced spine density (Strucinska et al., 2025). Interestingly, neurite mitochondrial morphology has been shown to be coupled to neuronal activity, including via activity-dependent regulation of MFF. High calcium levels in basal dendrites induced by neuronal signaling stimulated AMPK (AMP-activated protein kinase), which can phosphorylate and activate MFF to promote mitochondrial fission both in cultured hippocampal neurons (Hatsuda et al., 2023) and *in-vivo* (Virga et al., 2024). Furthermore, MFF has recently been shown to be locally translated in neurites, regulated by the RNA-binding protein FMRP (Fragile X messenger ribonucleoprotein), which associates with axonal and dendritic mitochondria in cultured hippocampal neurons, allowing local spatial regulation of mitochondrial morphology (Fenton et al., 2024). While no differences have been reported in peroxisomal morphology between different neuronal compartments (Wang et al., 2018)—perhaps since, unlike mitochondria, peroxisomes cannot undergo fusion (Bonekamp et al., 2012)—it is feasible that these compartment-specific differences in MFF/FIS1 could regulate the ability of peroxisomes to locally proliferate in response to signals. Whether this is the case, and whether this then impacts on neuronal and synaptic function (e.g., by altering membrane lipid composition or ROS signaling), remains an outstanding question.

### 2.2 Dysfunctional peroxisome dynamics in neurological disease

In humans, defects in peroxisome function, dynamics and/or biogenesis leads to a range of inherited metabolic disorders, such as Zellweger Syndrome and X-linked adrenoleukodystrophy (X-ALD), which notably present with neurological symptoms, amongst others. Furthermore, peroxisomes and peroxisomal metabolites are observed to be altered in a range of more common diseases, including age-related neurodegeneration (Zalckvar and Schuldiner, 2022). As well as defects in peroxisomal metabolism leading to pathophysiology, it is now becoming clear that dysfunctional peroxisome dynamics can also be a hallmark, or even a driver, of neurological diseases, as discussed here.

#### 2.2.1 Neurological symptoms of disorders of peroxisome dynamics

Patients have been reported with genetic defects in a number of proteins controlling peroxisome growth and division, including MFF, DRP1, GDAP1, and PEX11β, presenting with a range of symptoms including neurological and developmental abnormalities (reviewed in Carmichael et al., 2022). Importantly, where measured, these patients only display mild or no alterations to peroxisome metabolism, yet still display clinical symptoms (Ebberink et al., 2012; Koch et al., 2016; Hogarth et al., 2018), demonstrating that loss of peroxisome dynamics can lead to pathology independent of metabolic dysfunction, which emphasizes how crucial the ability of organelles to dynamically respond to their environmental is for human health (Carmichael et al., 2022).

Patients with mutations disrupting the function/expression of DRP1 and MFF would be predicted to have compromised peroxisomal and mitochondrial fission: accordingly, where investigated, fibroblasts from these patients typically display highly elongated mitochondria and peroxisomes, with peroxisomes being reduced in number (Waterham et al., 2007; Passmore et al., 2020). In both cases, the symptoms are predominately neurological—common symptoms of DRP1 mutations include encephalopathy, optic atrophy and epilepsy/seizures (Fahrner et al., 2016; Gerber et al., 2017; Whitley et al., 2018; Verrigni et al., 2019), while MFF-deficient patients present with symptoms including peripheral neuropathy and motor, speech and intellectual deficits (Shamseldin et al., 2012; Koch et al., 2016; Nasca et al., 2018; Panda et al., 2020). While this further highlights the importance of peroxisome/mitochondria dynamics in the brain, the exact pathophysiological mechanisms remain unclear, and the relative contribution of the peroxisomal vs. mitochondrial defects in these patients has not been addressed. A recent study, using cortical neurons derived from iPSCs from patients with dominant-negative DRP1 mutations, demonstrated aberrant synapse development and calcium signaling compared to controls, which could explain some of the observed symptoms (Baum et al., 2024). In patient-derived iPSCs, the hyper-elongated mitochondria are observed to be differentially trafficked in the axons, but whether this also applies to hyper-elongated peroxisomes has not been determined (Baum et al., 2024).

GDAP1 is predominantly expressed in neural cells, most strongly in neurons but also in oligodendrocytes, astrocytes and microglia (Huber et al., 2016; Karlsson et al., 2021). Mutations in GDAP1 are associated with Charcot-Marie-Tooth syndrome (CMT), a hereditary motor and sensory neuropathy, particularly affecting peripheral neurons (Bird, 1993). Although, like DRP1 and MFF, it plays a dual role in mitochondrial and peroxisome division, both organelles are not necessarily affected in patients carrying a GDAP1 mutation. Knockdown-rescue studies in a mouse neuroblastoma cell line showed that expression of disease-associated N-terminal mis-sense mutants of GDAP1 in GDAP1-silenced cells was able to restore peroxisomal fission, but not mitochondrial fission. However, GDAP1 mutants lacking an intact C-terminal domain were unable to rescue either peroxisome or mitochondrial morphology (Huber et al., 2013). Crucially, patients with C-terminal GDAP1 mutations display more severe clinical phenotypes (Kabzinska et al., 2011), indicating loss of peroxisomal dynamics makes a significant contribution to certain CMT pathologies. Furthermore, GDAP1 has also recently been proposed to play a role in peroxisome-mitochondria interactions (see section 3.2), which could also play a role in the axonopathy seen in GDAP1-associated CMT (Cantarero et al., 2024).

Confirming the importance of peroxisome dynamics for neuronal health, multiple PEX11β-deficient patients have been identified, presenting with neurological symptoms reminiscent of a mild peroxisomal biogenesis disorder, despite mainly normal biochemical parameters (Ebberink et al., 2012; Tian et al., 2020). Notably, while patients can display symptoms including intellectual disability, seizures and neuropathy (Malekzadeh et al., 2021; Tsinopoulou et al., 2023; Khoddam et al., 2024), one common feature amongst all patients is congenital cataracts and short stature, suggesting functional peroxisome dynamics are necessary for healthy development (Carmichael et al., 2022). Consistent with the role of PEX11β in the fission stage of peroxisome growth and division, PEX11β-deficient patient fibroblasts display a reduced number of peroxisomes compared to controls (Ebberink et al., 2012). Perhaps surprisingly, given PEX11β also mediates membrane deformation, PEX11β-deficient peroxisomes are also slightly elongated, which may indicate a compensatory function of one of the other PEX11 isoforms, likely PEX11γ (Ebberink et al., 2012). Interestingly, while loss of PEX11β, unlike DRP1 or MFF, would not be predicted to have a direct effect on mitochondrial dynamics, mislocalization of peroxisomal proteins to mitochondria has been observed in PEX11β-deficient patient cells as well as knock-out cell lines (Lismont et al., 2019; Carmichael et al., 2022). This indicates that a loss of peroxisome plasticity could have secondary effects on other organelles that could be contributing to the neurological pathophysiology.

Since all seven families identified so far with PEX11β-deficiency have mutations that would be predicted to abolish PEX11β expression (Malekzadeh et al., 2021; Tsinopoulou et al., 2023; Khoddam et al., 2024), studies using *Pex11*β knock-out mice are valuable for understanding the neuronal basis of the pathophysiology. Global *Pex11*β homozygous knock-out mice die shortly after birth but display decreased neuronal migration in the neocortex and increased apoptosis of neurons, despite only mild metabolic alterations and no structural changes to mitochondria (Li et al., 2002). Furthermore, as well as increased cell death, primary cortical cultures from these *Pex11*β knock-out mice show delayed development (see section 2.1.1) and higher ROS levels (Ahlemeyer et al., 2012), which could underlie the neurological symptoms observed in the patients. It is important to note, however, that the pathology of the *Pex11*β knock-out mouse is far more severe than the patients, who live longer and have not been reported to present with brain development defects at birth, likely reflecting species-specific differences (Carmichael et al., 2022).

#### 2.2.2 Changes to peroxisome dynamics in neurodegeneration

In addition to inherited disorders affecting peroxisome function and/or dynamics, it is increasing becoming clear that peroxisome dysfunction is observed in a broad range of more common neurological/neurodegenerative diseases, including Alzheimer’s Disease (AD), Parkinson’s Disease (PD), and multiple sclerosis (MS) (Zalckvar and Schuldiner, 2022). The metabolic profiles of these diseases are often characteristic of compromised peroxisome function, for example reduced plasmalogen and DHA levels in AD, increased ROS in PD, and VLCFA accumulation in MS (Fabelo et al., 2011; Kou et al., 2011; Gray et al., 2014; Deb et al., 2021). Several studies have correlated changes in peroxisome number and/or distribution, indicative of altered dynamics, with neurodegeneration, which could either represent a cause of pathophysiology, or a compensatory protective mechanism.

##### 2.2.2.1 Alzheimer’s disease

**FIGURE 3:**
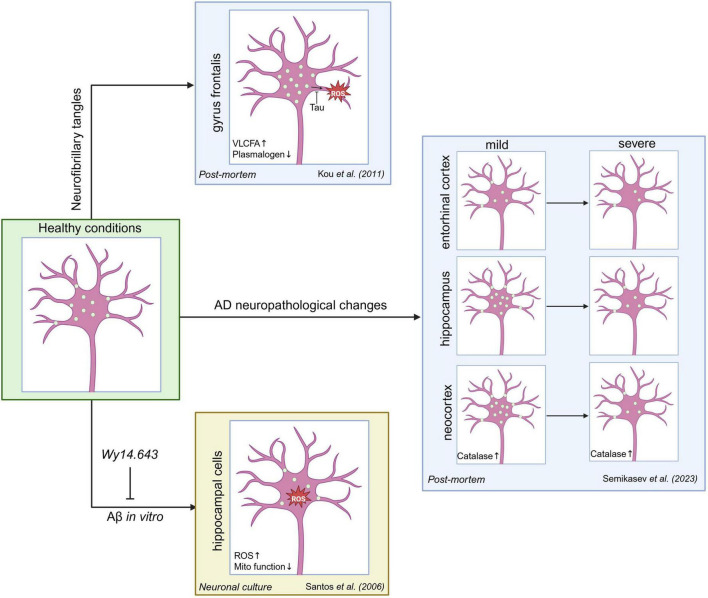
Changes in peroxisome density in Alzheimer’s disease. Schematic of reported alterations to numbers and distribution of peroxisomes (green circles) in different brain regions and AD pathologies. Peroxisome density in the soma is increased in gyrus frontalis neurons with neurofibrillary tangles, whereas there are a reduced number of peroxisomes in the neurites (Kou et al., 2011). It has been proposed that tau inhibits peroxisome transport into neurites, leading to oxidative stress (Stamer et al., 2002). In the entorhinal cortex, peroxisome density decreases as the severity of AD neuropathic changes (encompassing Aβ deposits, neurofibrillary tangles and neuritic plaques) increases (Semikasev et al., 2023). Conversely, the hippocampus and neocortex exhibit increases in peroxisome density in mild AD cases (accompanied by increased catalase expression in the neocortex) but decreases in severe cases (Semikasev et al., 2023). Exposure of cultured hippocampal neurons to Aβ fibrils reduces peroxisome density and increases ROS production and mitochondrial dysfunction. Pretreatment with the PPARα agonist Wy14.643 induces peroxisome proliferation and ameliorates the Aβ-induced cellular phenotypes including neurite loss and cell death. Created in BioRender (https://BioRender.com/51u41fa).

Using the peroxisomal membrane protein (PMP) PMP70 as a marker, peroxisomal density was assessed by immunofluorescence in the *post-mortem* gyrus frontalis from 30 patients with increasing progression of AD pathology. This demonstrated a significant increase in peroxisome density in the soma of neurons from patients with more advanced AD, but notably only in tissues with the pathological hallmark of neurofibrillary tangles (NFTs), consisting of hyperphosphorylated tau aggregates, not those with amyloid-beta (Aβ) plaques (Kou et al., 2011). This contrasted with peroxisome density in neurites, where peroxisomes were not detected in processes positive for phospho-tau (Figure 3). Mechanistically, elevation of tau, a microtubule-associated protein, has previously been shown to prevent peroxisome transport into neurites, which increased vulnerability to oxidative stress, leading to neurite degeneration (Stamer et al., 2002). Interestingly, while both mitochondria and peroxisomes were reduced in neurites with mature NFTs, only peroxisomes were affected in neurites showing milder pre-tangles, with mitochondrial distribution remaining normal, which may imply dysfunctional peroxisome distribution is an earlier pathological event (Kou et al., 2011). The loss of peroxisomes from distal neurites would be expected to compromise peroxisomal function in these processes, including ROS detoxification and delivery of synaptic/vesicle membrane precursors, which may contribute to the neurodegenerative phenotype (Semikasev et al., 2023). Indeed, this patient cohort also displayed metabolic alterations in the brain characteristic of peroxisome dysfunction, including VLCFA accumulation and reduced plasmalogen levels (Figure 3), though whether this is directly related to the changes in peroxisome dynamics is unknown (Kou et al., 2011).

A subsequent morphometric study in *post-mortem* human brain using PEX14 immunofluorescence as a marker reported differential changes to peroxisome density depending on brain region and degree of AD neuropathology. Whilst, on average, peroxisome density steadily decreased in the entorhinal cortex (affected early in the disease), in the hippocampus and neocortex it was observed to increase at early AD stages, then decrease as the disease progressed (Figure 3; Semikasev et al., 2023). The differences in peroxisome alterations between brain regions likely reflects differential sensitivity to disease, which could result from pre-existing differences in peroxisome function (e.g., antioxidant capacity) between areas (Chen et al., 1994). Notably, only in the neocortex was the increase in peroxisomes in mild AD patients accompanied by an increase in the number of catalase-positive peroxisomes (Figure 3), perhaps as an initial stress response to mitigate excess ROS production. Similar results were obtained in Tg2576 transgenic mice, which accumulate Aβ plaques—at 3 months, which represents early stage AD, peroxisomes were upregulated in both the hippocampus and neocortex, concomitant with signs of oxidative stress (Cimini et al., 2009). However, whether these changes in peroxisome number are protective or pathological remains to be seen, depending on whether they are contributing to or responding to ROS burden. It is important to note that different peroxisomal marker proteins display different profiles during AD progression, suggesting that peroxisome function is likely changing over the disease course, as well as number (Fanelli et al., 2013; Semikasev et al., 2023). Since inhibition of peroxisomal β-oxidation in cultured rat cortical neurons increased Aβ production, it could be that AD-induced peroxisome dysfunction initiates a “vicious cycle” to accelerate disease pathology (Shi et al., 2012). Overall, despite differences depending on brain region, disease stage and pathological mechanism, it seems that alterations in neuronal peroxisome density, and concomitant dysregulation of peroxisomal function, may represent a common feature of AD (Figure 3).

Whilst the stimuli and mechanisms leading to the observed changes in peroxisome abundance are not clear, it is notable that multiple studies have reported changes in the expression or activity levels of proteins controlling peroxisome growth and division in AD patients and models. While most studies have observed an increase in DRP1 expression/GTPase activity in *post-mortem* AD brains compared to controls (Manczak et al., 2011), decreases have also been reported (Wang et al., 2009), which may reflect differences in brain region, disease stage and/or pathological mechanism (Bera et al., 2022). Furthermore, while treatment of primary rat cortical neurons with Aβ *in-vitro* decreased DRP1 and MFF protein levels in one study (Soares et al., 2022), other studies have observed activation of mitochondrial fission via an increase in AMPK-dependent MFF stimulation in both *in-vitro* AD models (Lee et al., 2022) and human post-mortem brain tissue (Fang et al., 2019). An unusual “mitochondria-on-a-string” morphology, resulting from stalled fission despite DRP1 recruitment, has also been observed in both *post-mortem* AD brains and transgenic mouse models, attributed to reduced DRP1 GTPase activity (Zhang et al., 2016). As previously discussed (see section 2.1.3), these changes are typically interpreted through their effects on mitochondrial dynamics, though it is highly likely they would also impact peroxisome proliferation, and could be underpinning the changes in peroxisome density seen in AD. However, regardless of the mitochondrial vs. peroxisomal contributions, the role played by altered organelle dynamics in disease progression *in-vivo* remains to be seen.

##### 2.2.2.2 Parkinson’s disease

Much like AD, peroxisomal metabolites are altered in PD patients, for example reduced plasmalogen levels were observed in the frontal cortex *post-mortem* (Fabelo et al., 2011). Peroxisomal dysfunction also exacerbates the pathology of α-synuclein, the accumulation and deposition of which is a hallmark of PD. In mouse models of peroxisome biogenesis disorders, where peroxisome function is lost, α-synuclein oligomerization was increased, leading to altered fatty acid profiles (Yakunin et al., 2010). However, whether changes to peroxisome abundance occur during PD disease progression has not been investigated. A role for pexophagy in PD pathology has recently been proposed, with HSPA9 (heat shock protein A9/mortalin) identified as a negative regulator of pexophagy. Silencing of HSPA9 in neuroblastoma SH-SY5Y cells induced a decrease in peroxisome number via the canonical autophagy pathway, as a result of increased ROS levels (Jo et al., 2020). Importantly, HSPA9 levels are reportedly reduced in AD and PD, and HSPA9 loss-of-function mutations have been found in PD patients (Burbulla et al., 2010). Accordingly, expression of PD-associated HSPA9 mutants were not sufficient to inhibit pexophagy in HSPA9-depleted cells (Jo et al., 2020). Altogether, while pexophagy is an important quality control mechanism, this study suggests excess pexophagy, triggered by increased ROS burden, could enhance neuronal damage in PD.

##### 2.2.2.3 Other neurodegenerative diseases

Another neurodegenerative disease bearing the hallmarks of peroxisomal dysfunction (in this case, VLCFA accumulation in gray matter) is MS. *Post-mortem* gray matter from MS patients showed a reduced number of PMP70-positive neurons relative to controls, which negatively correlated with disease duration (Gray et al., 2014). MS symptoms notably overlap with X-ALD, where VLCFA import into peroxisomes is compromised but dynamics are normal. Given that fibroblasts from patients with PMP70 deficiencies exhibit normal rates of β-oxidation of the VLCFA C26:0 (Ferdinandusse et al., 2015), it is probable that the reduction in PMP70-positive neurons in MS reflects an overall decrease in peroxisome number/function, which limits the rate of peroxisomal β-oxidation and exacerbates the inflammation and demyelination phenotypes via VLCFA accumulation. Similar to the other neurodegenerative diseases mentioned, the mechanism by which peroxisomes are reduced in MS is unclear, but is likely a secondary effect of initial inflammation/stress (Gray et al., 2014).

As previously discussed in relation to AD (see section 2.2.2.1), changes in abundance are not the only alterations to peroxisome dynamics seen in neurodegeneration—differences in peroxisome trafficking and distribution are also observed. Hereditary spastic paraplegia (HSP), characterized by lower-limb spasticity and paralysis, arises from axonal loss, particularly in the corticospinal tract. The most common cause of autosomal-dominant, adult-onset HSP is mutations in spastin, a protein which severs stabilized microtubules and is therefore required for transport of organelles including mitochondria and peroxisomes (Abrahamsen et al., 2013). Differentiated olfactory neurosphere-derived stem cells from patients with spastin mutations had fewer peroxisomes along their axons compared to healthy controls, and the peroxisomes moved more slowly on average and were more likely to be immobile. Differentiated patient cells also displayed higher expression of oxidative stress markers, which could be reduced by the microtubule stabilizing drug epothilone D, implying defective microtubule-dependent peroxisomal (and potentially mitochondrial) transport results in a redox imbalance that in turn leads to axonal degradation (Wali et al., 2016). Since neuronal cells become more sensitive to H_2_O_2_ treatment when peroxisome trafficking into neurites is compromised by tau overexpression (Stamer et al., 2002), dysfunctional peroxisome distribution across neuronal compartments could be a common mechanism exacerbating the damaging effects of oxidative stress that underlies a number of neurodegenerative disorders.

#### 2.2.3 Modulating peroxisome dynamics as a therapeutic target for neurological disease/neurodegeneration

Oxidative damage, as measured by RNA and protein modification, is one of the earliest signs of pathology in human AD patients (Nunomura et al., 2001), making it a plausible point-of-intervention before irreversible neurodegeneration occurs. The ability of peroxisomes to detoxify H_2_O_2_ (see section 2.1.2) confers a protective effect in cellular models of AD, with catalase inhibition exacerbating the toxicity of exogenous Aβ fibrils when added to rat hippocampal neuronal cultures (Santos et al., 2005). Therefore, increasing the antioxidant capacity of peroxisomes through upregulating their proliferation could represent an attractive therapeutic strategy for AD (Figure 3). Pretreatment of rat hippocampal neurons with the PPARα activator Wy14.643 increased the number of peroxisomes per neuronal cell and ameliorated the pathophysiological effects of Aβ fibrils, including reducing Aβ-induced ROS generation, neurite loss and cell death, and increasing mitochondrial viability, compared to cells without Wy14.643 treatment (Figure 3; Santos et al., 2005). Consistent with this protective effect, treatment of transgenic AD model mice *in-vivo* with Wy14.643 as well as another peroxisome proliferator, 4-phenylbutyric acid, improved spatial and recognition memory and protected synaptic function (Inestrosa et al., 2013). However, since the oral administration of these compounds will lead to systemic effects (e.g., inducing liver and kidney peroxisome metabolism), it is possible that some aspects of their protective effects on memory are secondary, as a result of increased supply of important liver-synthesized lipids such as DHA and plasmalogens to the brain (Darwisch et al., 2020; Wang et al., 2020). Additionally, since peroxisomes in human cells display a poor proliferative response to PPAR agonists, compared to rodent cells (Lawrence et al., 2001), other strategies for upregulating peroxisome number may have to be adopted to generate a viable therapy for human AD patients.

DHA is an omega-3 polyunsaturated fatty acid (PUFA) that requires peroxisomal β-oxidation for its synthesis, with roles in neuron/synapse development and function (Dyall, 2015). Data from various animal models of AD/aging suggest DHA is neuroprotective, while in patients high DHA dietary/plasma levels are associated with a lower risk of AD (Belkouch et al., 2016). Notably, DHA has also been shown to stimulate peroxisome proliferation under conditions where peroxisomes are reduced in number (in this case, in fibroblasts with peroxisomal β-oxidation deficiencies), in a DRP1-dependent manner. Mechanistically, DHA seems to promote the elongation phase of growth and division by enhancing PEX11β oligomerization on the peroxisomal membrane (Itoyama et al., 2012). While DHA likely mediates a number of beneficial pleiotropic effects (Belkouch et al., 2016), it is possible that restoration of peroxisome number through DHA-mediated proliferation could be contributing to its neuroprotective function.

## 3 Peroxisome-organelle interplay in neuronal cells

While each organelle within the cell compartmentalizes distinct functions, it has now become clear that these compartments are not isolated entities, but instead cooperate with each other as part of an inter-organellar communication network that is essential to maintain cellular function (Silva et al., 2020). Peroxisomes are no exception, and must cooperate with a number of organelles such as the ER, mitochondria, lysosomes and lipid droplets to fulfill their roles in lipid and redox homeostasis (Schrader et al., 2020). One way this functional interplay can be mediated is via physical interactions at membrane contact sites (MCSs), where apposing organelle membranes are brought to within ∼10–30 nm of each other via the interaction of tethering proteins on one organelle with tethering proteins or membrane lipids on the other (Scorrano et al., 2019).

Although best characterized in yeast (Shai et al., 2016), peroxisome-organelle interactions are increasingly being studied in animal/mammalian cells, as their widespread importance for cellular physiology becomes appreciated (Sargsyan and Thoms, 2019; Schrader et al., 2020). The implications of some of the most established examples of peroxisome-organelle interplay, both functionally and physically, on neuronal cell function in health and disease, are discussed below.

### 3.1 Peroxisome-ER cooperation

**FIGURE 4:**
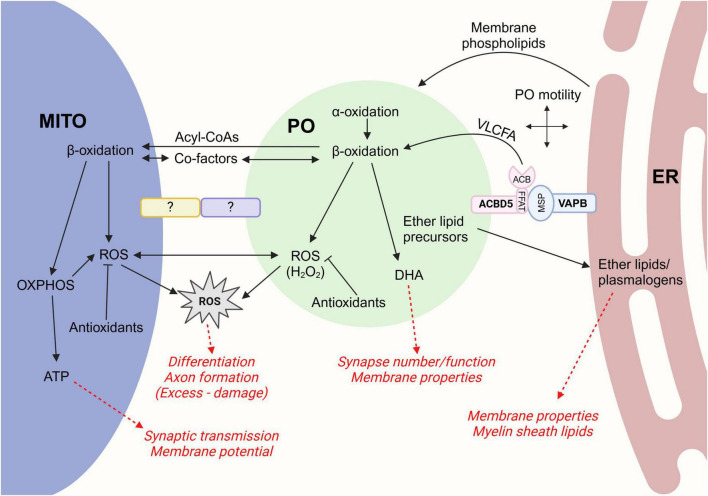
The effects of peroxisome-ER and peroxisome-mitochondria cooperation on neuronal function. Schematic of the interplay between peroxisomes-mitochondria and peroxisomes-ER. Physical interactions between peroxisomes and the ER can be mediated by the tether proteins ACBD5 and VAPB, while the peroxisome-mitochondria tethers are less clear. The impact on these cooperative processes on neuronal function are indicated in red. MITO, mitochondria; OXPHOS, oxidative phosphorylation; PO, peroxisome. Created in BioRender (https://BioRender.com/evm7dle).

Close connections between peroxisomes and the ER were first observed in early electron micrographs (Kalmbach and Fahimi, 1978; Zaar et al., 1987), and it is now known that peroxisomes interact with the ER more extensively than they do with any other organelle in a range of cell types including neurons (Valm et al., 2017; Rhoads et al., 2024)—for example, around 65% of peroxisomes are in direct contact with the ER in COS-7 cells, as assessed by EM (Costello et al., 2017a). As organelles that are key for lipid metabolism, peroxisomes and the ER cooperate in several biosynthetic pathways (Figure 4), including the production of ether-phospholipids and PUFAs (including DHA, see section 2.2.3), both of which are particularly relevant for neuronal function (Schrader et al., 2020). Plasmalogens, a class of ether-phospholipids, which begin synthesis in the peroxisome before being completed in the ER, are important components of intracellular and synaptic membranes as well as myelin sheath lipids. Highlighting their crucial roles in the brain, defects in ether-phospholipid synthesis result in severe neurological symptoms such as seizures and cerebellar atrophy, while plasmalogen levels have been observed to be reduced in a number of common neurodegenerative diseases (see section 2.2.2) (Dorninger et al., 2017).

Physical interactions between peroxisomes and the ER at MCSs facilitate their cooperative functions. Peroxisome-ER MCSs are mediated by the tail-anchored tethering protein ACBD5 (acyl-CoA binding domain protein 5) in the peroxisomal membrane [and, to a lesser extent, ACBD4 (Costello et al., 2017b)], which interacts via its FFAT (two phenylalanines in an acidic tract)-like motif with the MSP (major sperm protein) domain of the ER-resident VAP (vesicle-associated membrane protein-associated protein) proteins A/B (Figure 4; Costello et al., 2017a; Hua et al., 2017). Evidence that ACBD5 plays important roles in the brain comes from *Acbd5* knock-out mice, which display a progressive degeneration of the cerebellum, presenting as symptoms of ataxia such as kyphosis and unsteady gait. While there was no difference in synapse density or distribution observed in mice lacking ACBD5, cerebellar Purkinje cells were reported to be reduced in number, and displayed more axonal swellings, accompanied by organelle accumulation in these regions (Darwisch et al., 2020; Granadeiro et al., 2024). However, since ACBD5 also plays a role in capturing VLCFAs via its acyl-CoA binding domain for import into peroxisomes, it is unclear to what extent the *Acbd5* knock-out mouse phenotypes are a direct result of reduced peroxisome-ER tethering, versus a peroxisomal β-oxidation defect.

In addition to regulating lipid metabolism, peroxisome-ER MCSs also impact on peroxisome dynamics (Figure 4). Reduction of peroxisome-ER tethering via ACBD5 or VAPB silencing abrogated the peroxisome elongation seen in MFF-deficient patient cells, which importantly could be rescued by an artificial peroxisome-ER tether, indicating the proximity to the ER supports membrane expansion during peroxisomal growth and division, likely by providing the necessary phospholipids (see section 2) (Costello et al., 2017a). Interestingly, mutations in the lipid-transfer protein VPS13D (vacuolar protein sorting 13D), which mediates peroxisome biogenesis and localizes to multiple MCSs including peroxisome-ER, lead to neurological disorders with ataxia phenotypes (Baldwin et al., 2021; Guillén-Samander et al., 2021). Furthermore, loss of ACBD5 significantly increased peroxisome movement in human fibroblasts, suggesting attachment to the ER restricts peroxisome motility and can therefore influence their distribution (Costello et al., 2017a).

The role of ACBD5-dependent tethering to the ER on peroxisome trafficking within neuronal compartments was investigated in cultured hippocampal neurons. Under basal conditions, peroxisomes labeled with a fluorescent matrix marker were observed to be frequently associated with ER tubules in both the soma and neurites by super-resolution microscopy (Wang et al., 2018). Overexpression of ACBD5 reduced both the maximum and average speed of peroxisome movement in neurites, supporting the idea that peroxisome-ER tethering restricts motility. In terms of peroxisome distribution within the neuronal compartments, ACBD5 overexpression increased the proportion of peroxisomes in neurites, and resulted in redistribution of somatic peroxisomes toward the cell membrane. Notably, the same redistribution was observed upon overexpression of a FFAT-mutated ACBD5, suggesting this phenotype is independent of VAPB- and thus ER-binding, implying there may be additional factors interacting with ACBD5 to control peroxisome positioning in mammalian neurons (Wang et al., 2018). Conversely, depletion of the *Drosophila melanogaster* homolog of ACBD5, which also possesses a functional FFAT motif and tethers peroxisomes to the ER, led to an increase in the number of peroxisomes in the axons of both proximal and distal wing neurons (Kors et al., 2024). Since the same phenotype was observed when the VAPB homolog was depleted, this suggests peroxisome-ER MCSs regulate peroxisomal distribution in fly neurons, which may point to species and/or cell type differences between the functions and mechanisms of peroxisome-ER interactions.

### 3.2 Peroxisome-mitochondria cooperation

Peroxisomes and mitochondria, both being oxidative and metabolic organelles, perform cooperative tasks that necessitate contact for transfer of signals/intermediates (Figure 4). For example, peroxisomes need to (1) break down branched chain fatty acids by α-oxidation, and (2) chain-shorten VLCFAs by β-oxidation, before they can be further oxidized in mitochondria to generate energy. As well as the transfer of acyl-CoA intermediates, this requires a redox shuttle system between the two organelles to regenerate the co-factors required for these processes (Wanders et al., 2016). Furthermore, both organelles contribute to ROS homeostasis by producing and scavenging ROS (Lismont et al., 2015), and also share roles in steroid biosynthesis (Fan et al., 2016) and antiviral responses (Ferreira et al., 2022). Multiple proteins are targeted/recruited to both peroxisomes and mitochondria, including MFF, DRP1, FIS1, and GDAP1 (see section 2) (Costello et al., 2018), which may facilitate coordinated communication. Furthermore, roles shared by peroxisomes and mitochondria regulate the function of neurons. For example, cooperative break-down of α/β-oxidation substrates is required to maintain ATP generation and Ca^2+^ homeostasis in the brain (Schönfeld and Reiser, 2016), and physiological ROS signaling is involved in neuronal development, differentiation, firing and axon formation (Diano et al., 2011; Doser and Hoerndli, 2021), while excess ROS leads to lipid peroxidation, affecting neuronal membrane fluidity and conductance which disrupts excitatory signaling (Figure 4; Tracey et al., 2018).

Remarkably, neurons, or more specifically neurites, show considerably more extensive peroxisome-mitochondrial juxtaposition than other cells investigated, with 80% of peroxisomes in contact with mitochondria, vs. 20% in COS-7 cells (Figure 2B; Valm et al., 2017; Wang et al., 2018), suggesting that direct interactions between the two organelles may be particularly crucial for healthy neuronal function. Indeed, by live imaging of mouse hippocampal neuronal cultures, peroxisomes have been observed to “surf” along mitochondria while maintaining contact, demonstrating their intimate physical connection which may support their cooperative function and/or coordinate their distribution in neurites. Overall, both peroxisomes and mitochondria remain fairly static in neurites, though peroxisomes exhibit more short-range movements, with both organelles moving at speeds consistent with microtubule-dependent motility (Wang et al., 2018). Supporting an important role for these contacts in neurons specifically, a recent study observed more peroxisome-mitochondria colocalization in rat cortical neurons compared to astrocytes, with these interactions persisting for longer. Crucially, neuronal peroxisome-mitochondria interactions were increased in response to arsenic treatment, suggesting these contacts may be protective against redox stress (Rhoads et al., 2024). How these interactions are mediated, and whether they regulate other peroxisome-mitochondria shared functions, is still unknown. Several potential peroxisome-mitochondria MCS tethers/regulators have been reported in mammals, including ABCD2/ECI2 (enoyl-CoA δ-isomerase 2), MFN1/2 (mitofusin 1/2) and GDAP1 (Fan et al., 2016; Huo et al., 2022; Cantarero et al., 2024), with their relative contributions and physiological roles in peroxisome-mitochondria communication in neurons becoming a burgeoning area of research.

### 3.3 Peroxisome-lysosome cooperation

As with the ER, the crucial role of peroxisomes and lysosomes in maintaining cellular lipid homeostasis necessitates cooperation between the two organelles. Physical peroxisome-lysosome contacts, mediated by the lysosomal membrane protein synaptotagmin 7 (Syt7) interacting with PI(4,5)P_2_ phospholipids in the peroxisomal membrane, are best characterized for their function in intracellular cholesterol transport (Jin et al., 2015). Via these contacts, peroxisomes are proposed to act as a shuttle to transport internalized cholesterol, which is initially routed to lysosomes, to the ER, where it can be further metabolized for structural and regulatory functions (Chu et al., 2015; Xiao et al., 2019). Dysfunction of this pathway has been suggested to explain the intracellular cholesterol accumulation observed in numerous inherited peroxisomal disorders, including Zellweger Syndrome (Chu et al., 2015).

Disruption of the interplay between peroxisomes and lysosomes has been implicated in the pathophysiology of peripheral neuropathy. Mice lacking functional peroxisomes in Schwann cells (through cell type-specific knock-out of *Pex5*, an essential peroxin for matrix protein import), displayed reduced conduction in peripheral nerves, in the absence of demyelination. Instead, distribution of the potassium channel K_*v*_1.1 within the axonal membrane was disrupted, localizing to ectopic patches in addition to its usual juxtaparanodal positioning. This coincided with an increased level of gangliosides (important axonal membrane lipids) which were observed to accumulate in enlarged puncta positive for lysosomal markers (Kleinecke et al., 2017). Together, this suggests a pathophysiological mechanism whereby peroxisomal dysfunction leads to a secondary lysosomal impairment, preventing the degradation of gangliosides which leads to their accumulation and the disruption of axonal membrane composition and function. Interestingly, lysosomal dysfunction has also been observed as a consequence of compromised peroxisomal β-oxidation in retinal pigment epithethial cells, resulting in an inability to digest VLCFA-rich photoreceptor outer segments and leading to retinal degeneration (Kocherlakota et al., 2023).

### 3.4 Dysfunctional peroxisome-organelle cooperation in disease

Our understanding of dysfunctional inter-organelle communication in neurological disease is so far centered around mitochondria-ER interactions, which are the best characterized MCSs in neurons (Markovinovic et al., 2022). Mitochondria-ER MCSs have been reported to be altered in a number of neurogenerative conditions including Motor Neuron Disease (Rickman et al., 2020), AD (Lau et al., 2020), Amyotrophic Lateral Sclerosis (ALS) (Lau et al., 2018) and PD (Gómez-Suaga et al., 2018). This compromised mitochondria-ER cooperation could be directly driving disease progression, due to the reported functions of these contacts, e.g., in maintaining Ca^2+^ homeostasis and regulating synapse number/activity in neurons (Bernard-Marissal et al., 2018; Gómez-Suaga et al., 2019). Interestingly, phenotypes consistent with loss of peroxisome-organelle shared functions are seen in a range of age-related and neurodegenerative disorders, including ROS imbalance and VLCFA accumulation (peroxisome-mitochondria; Nunomura et al., 2001; Kou et al., 2011; Pascual-Ahuir et al., 2017); reduced plasmalogen levels (peroxisome-ER; Dorninger et al., 2017); and dysfunctional cholesterol metabolism (peroxisome-lysosome; Dai et al., 2021). However, if or how physical connections via MCSs between peroxisomes and other organelles are altered in neurodegenerative diseases, and whether this may contribute to pathophysiology, remains to be seen.

Around 16 patients have now been identified with mutations affecting the peroxisome-ER tether protein ACBD5. Like other disorders affecting peroxisomes, the reported symptoms are mainly progressive and neurological, including ataxia, cognitive decline and seizures (Carmichael et al., 2022; Dawes et al., 2025). Notably, the patients exhibit a mild elevation in plasma VLCFA levels while all other peroxisomal metabolites are normal, consistent with a role of ACBD5 in the peroxisomal β-oxidation pathway, likely capturing VLCFAs via its ACB domain at the peroxisome-ER interface to facilitate their import (Figure 4; Schrader et al., 2020). However, given the subtle phenotypic differences between ACBD5-deficient patients compared to those with VLCFA import defects (e.g., X-ALD), it is plausible that loss of ACBD5’s direct peroxisome-ER tethering role could be impacting on the pathophysiology, e.g., through changes to peroxisome dynamics/distribution or cooperative peroxisome-ER lipid metabolism. Furthermore, the ER-resident component of the peroxisome-ER tether, VAPB, has been linked to a variety of neurological disorders, most notably ALS (Kors et al., 2022). ALS Type 8 is a familial form of the disease presenting with neurological symptoms including limb weakness, cognitive impairment and tremors (McBenedict et al., 2025), and is associated with a point mutation in the MSP domain of VAPB which is reported to reduce its affinity for FFAT motif-containing proteins (Teuling et al., 2007). The mutated VAPB protein is prone to aggregation and leads to peroxisome clustering around the ER when overexpressed, which could be reduced by ACBD5 knock-down (Hua et al., 2017). While this disruption to peroxisome-ER contact may be contributing to the ALS8 phenotypes, this must be interpreted with caution, since VAPB mediates ER interactions with multiple other organelles, including mitochondria, endosomes, Golgi and the plasma membrane, meaning the symptoms likely arise from a broad loss of ER cooperation with other cellular compartments (Kors et al., 2022).

## 4 Conclusion

From the discovery of the first peroxisomal disorders, the crucial roles played by this small but mighty organelle in many aspects of brain development and function have been apparent (Berger et al., 2016). While the metabolic functions of peroxisomes are well-established to be essential in the nervous system, it is now becoming clear that other facets of peroxisome biology, including their regulation, plasticity, dynamics and interactions, are also key determinants of neuronal health and disease (Carmichael et al., 2022). Much of the current evidence for this comes from the bidirectional correlation between altered peroxisome dynamics/interactions and neurological dysfunction—therefore, a key focus for future research is to reveal the molecular mechanisms by which these aspects of peroxisomal biology regulate physiological and pathophysiology neuronal processes. A better understanding of the roles that peroxisome dynamics and inter-organelle interactions play in neuronal health and disease may inspire future therapies to treat neurological and neurodegenerative disorders, currently a major burden globally.

### 4.1 Outstanding questions on the role of peroxisome dynamics/inter-organelle interactions in physiological neuronal function

Compared to mitochondrial dynamics, our understanding of how dynamic peroxisome behavior facilitates physiological neuronal processes is in its infancy. For example, it is unclear if peroxisomes play a direct role in neurotransmission, despite regulating (synaptic) membrane composition and properties. Peroxisomes have also been proposed to play a role in excitation-dependent intracellular calcium dynamics in cardiomyocytes (Sargsyan et al., 2021); while not yet studied in neurons, this may suggest another mechanism by which peroxisomes could modulate neurotransmission. Notably, Ca^2+^ exchange at mitochondria-ER MCSs facilitates synaptic transmission by promoting mitochondrial ATP production (see section 4.3) (Paillusson et al., 2017; Gómez-Suaga et al., 2019), but it has yet to be shown if peroxisome-mitochondria/ER MCSs can mediate calcium signaling between organelles.

Conversely, are peroxisome dynamics and/or organelle interactions activity-dependent? Given the plasticity of the peroxisomal compartment, it is plausible that neuronal activity could stimulate a peroxisomal response, e.g., inducing proliferation to increase metabolic capacity. Furthermore, while the shared peroxisome/mitochondria fission machinery components DRP1 and MFF have been shown to regulate neuronal function in an activity-dependent manner (see section 2.1.3), whether this impacts peroxisome dynamics, and, if so, whether this contributes to the observed phenotypes, needs to be investigated. Additionally, while peroxisomes are distributed throughout neuronal compartments including the soma and neurites (Figure 2A; Wang et al., 2018), the functional significance of these local peroxisome populations is still unknown.

So far, research into peroxisome-organelle cooperation in mammalian cells has mainly focused on the fundamental mechanisms and functions of these interactions, typically in cell lines (Silva et al., 2020). Given the abundance of peroxisome-mitochondria interactions in neurons (Figure 2B; Wang et al., 2018), understanding the roles of this organelle communication in neuronal (and, moreover, glial) cells is a key area for future investigation. Additionally, while the shared functions of peroxisomes and mitochondria in β-oxidation and ROS homeostasis are likely to be important for neuronal physiology, whether this cooperation requires physical contacts via MCSs and, if so, how these MCSs are formed and regulated, has yet to be determined. Understanding how specific peroxisome-organelle MCSs contribute to neuronal health and disease necessitates creative experimental strategies to manipulate one type of MCSs with minimal effect on the others, which is a challenge given the interconnectivity of the organellar network, and the presence of certain tether proteins (e.g., VAPB) at multiple MCSs (Kors et al., 2022).

### 4.2 Outstanding questions on the role of peroxisome dynamics/inter-organelle interactions in neurological disease

The observation that peroxisomes and/or peroxisomal metabolites are altered in such a broad range of neurological and neurodegenerative disorders suggests they may represent a common pathophysiological mechanism, placing them as promising therapeutic target to treat a spectrum of age-related diseases affecting the brain. More broadly, peroxisome dysfunction, for example as a result of reduced density, could plausibly be a contributing factor to the oxidative stress and wide-ranging dysregulation of lipid metabolism considered to be hallmarks of neurodegenerative pathologies (Zhao et al., 2023; Houldsworth, 2024). However, whether changes to peroxisome dynamics and/or interactions contribute to the pathophysiology, or are instead a compensatory response, is as yet unclear. While increasing peroxisome number via stimulating proliferation has been proposed as a protective strategy to counteract increased redox in neurodegeneration (Santos et al., 2005), this is potentially a double-edged sword due to the peroxisome’s ability to both produce and detoxify ROS (Fransen et al., 2012). Furthermore, studies proposing a beneficial role of peroxisome proliferators in neurodegenerative conditions have used rodent models (Santos et al., 2005; Inestrosa et al., 2013), which respond much better to such compounds than human cells (Lawrence et al., 2001)—therefore a better understanding of the regulation of peroxisome dynamics in humans is important for this to be a viable therapeutic option.

Surprisingly, patients with deficiencies in proteins affecting peroxisome dynamics (e.g., MFF, PEX11β) exhibit neurological symptoms despite little or no alterations to the levels of peroxisomal metabolites, in contrast to diseases where peroxisomal function is impaired, such as peroxisome biogenesis disorders (Carmichael et al., 2022). While this indicates that the ability of peroxisomes to respond appropriately to cellular changes, independent of metabolism, is important in the brain, the pathophysiological mechanisms are not fully elucidated. Despite the elongated nature of peroxisomes in these patients, they can still be degraded by autophagic processes (Passmore et al., 2020), however their transport, e.g., into thin neurites may be compromised (Schrader et al., 2014). Furthermore, the inability to divide leads to a reduction in peroxisome number (Passmore et al., 2020), which may impact on their communication with other organelles. It may also be the case, since peroxisomal metabolites are measured in plasma and/or fibroblasts for diagnostic purposes, there may be as-yet unnoticed metabolic alterations in the neuronal tissue of these patients, where extremely long VLCFAs are naturally found (Ferrero et al., 2025). Alternatively, these elongated peroxisomes may trigger alternative metabolic or signaling pathways that are not currently analyzed in classic biochemical screens.

Although phenotypes consistent with loss of peroxisome-organelle interplay are observed in a range of neurological diseases, whether physical peroxisome-organelle interactions via MCSs are directly or indirectly altered in these conditions, either in models or in patients, has yet to be investigated. Combined with a better understanding of the physiological functions and regulation of these contacts, targeting organelle communication networks could be a future strategy for improving neuronal performance in these conditions.
